# Seasonal Deposition and Lifting of Dust on Mars as Observed by the Curiosity Rover

**DOI:** 10.1038/s41598-018-35946-8

**Published:** 2018-12-04

**Authors:** Á. Vicente-Retortillo, G. M. Martínez, N. Renno, C. E. Newman, I. Ordonez-Etxeberria, M. T. Lemmon, M. I. Richardson, R. Hueso, A. Sánchez-Lavega

**Affiliations:** 1 Department of Climate and Space Sciences and Engineering, University of Michigan, Ann Arbor, MI USA; 2 Aeolis Research, Pasadena, CA USA; 3 Departamento de Física Aplicada I, Universidad del País Vasco, Bilbao, Spain; 4 Space Science Institute, College Station, TX USA

## Abstract

*In situ* measurements by the Curiosity rover provide a unique opportunity for studying the effects of dust on assets placed at the surface of Mars. Here we use *in situ* measurements of solar UV radiation to quantify the seasonal and interannual variability of dust accumulation on the sensor on the rover deck. We show that the amount of dust accumulated on the sensor follows a seasonal cycle, with net dust removal during the perihelion season until L_s_ ~ 300°, and net dust deposition until the end of the aphelion season (L_s_ ~ 300°–180°). We use independent *in situ* measurements of atmospheric opacity and pressure perturbations in combination with numerical modeling, showing that daytime convective vortices and nighttime winds are likely responsible for the seasonal dust cleaning, with the role of nighttime wind being more important in Martian Year (MY) 32 than in MY 33 and that of daytime convective vortices being more important in MY 33 than in MY 32. The fact that the UV sensor is cleaner in MY 33 than in MY 32 indicates that natural cleaning events make solar energy an excellent candidate to power extended (multiannual) Mars missions at similar latitudes as the Curiosity rover.

## Introduction

Airborne dust is well known to pose serious challenges to the exploration of Mars, particularly during the dust storm season. Now that not only government agencies, but also private industry are interested in establishing long-lasting infrastructures at the surface of Mars, it becomes extremely important to understand the effect of dust on assets placed at its surface.

Airborne dust is ubiquitous throughout the year, but its amount varies seasonally^1–6^ as a result of complex feedbacks between dust lifting from the surface, transport, and deposition, with the evolving atmospheric circulations^7–12^.

**Figure 1:**
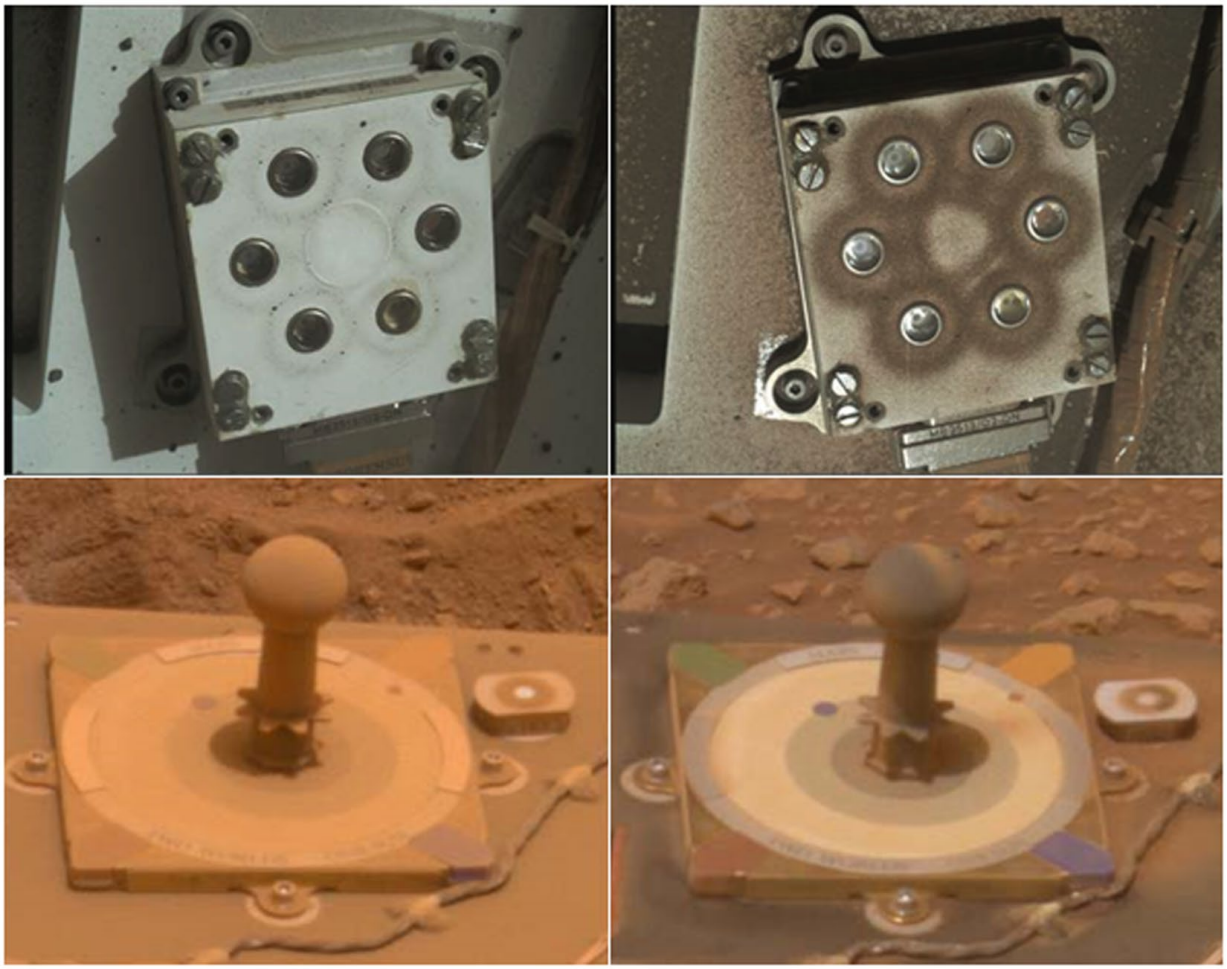
(Top) MAHLI images of the REMS UV Sensor on sols 36 (left) and 1314 (right) of the MSL mission; dust patterns are caused by circular magnetic rings (PDS images 0036MH0000540010100063E01 and 1314MH0000950010500447C00). (Bottom) PanCam images of the MER-A calibration target on sols 416 (left) and 426 (right) of the MER-A mission (image credit: NASA/JPL/Cornell, https://mars.nasa.gov/system/downloadable_items/38385_Sol416A_426A_cal_targets-A433R1.jpg).

Several solar-powered missions have operated successfully at the Martian surface, such as the Mars Pathfinder (MPF) Sojourner rover and Carl Sagan Memorial Station, the Mars Exploration Rovers Spirit (MER-A) and Opportunity (MER-B), and the Phoenix (PHX) Lander. The periodical removal of dust from their solar panels (dust removal from MER-A is shown in Fig. 1, bottom panel) has permitted Spirit and Opportunity to operate for more than 2,000 and 5,000 sols (Martian days), despite their planned duration of only 90 sols.

Data from the MER solar panels have been used to study dust accumulation and removal at their locations^13,14^. Unfortunately, neither the data from the solar panels of the MER rovers, nor that from any other solar-powered surface-based Mars spacecraft, are available in public archives. Moreover, the MER rovers did not carry a dedicated meteorological package and therefore the dust lifting mechanisms responsible for cleaning their solar panels could not be unveiled using independent sets of *in situ* data.

The Mars Science Laboratory (MSL) Curiosity rover landed at Gale Crater (4.6° S) in August of 2012^15^ carrying scientific instruments that allow not only the quantification of variations in the amount of dust accumulation on its deck, but also the study of dust deposition and lifting mechanisms responsible for such variations using publicly-available data. In particular, the Rover Environmental Monitoring Station (REMS) contains a suit of sensors to measure pressure, air and ground temperatures, wind speed and, for the first time, ultraviolet (UV) radiation in six bands between 200 and 380 nm^16^. Due to its placement on the rover deck, the UV sensor is extremely useful for studying dust accumulation and removal by atmospheric processes. In addition, Curiosity is equipped with the Mast Camera (Mastcam), which can be used to measure aerosol opacities from images of the Sun^17^, and the Mars Hand Lens Imager (MAHLI) camera^18^, which has been used to monitor dust accumulation on the rover surfaces, such as the UV sensor (Fig. 1, top panel).

Here we have used REMS UV measurements in combination with atmospheric opacities retrieved from Mastcam images to quantify the seasonal and interannual variability of dust accumulation on the UV sensor over the first 1648 sols (~2.5 Martian Years (MY)) of the mission. We have also used REMS pressure measurements in combination with results from the MarsWRF mesoscale model^19–21^ to shed light on the dust lifting mechanisms responsible for the observed variability.

## Seasonal and Interannual Variability of Dust Accumulation and Removal

**Figure 2:**
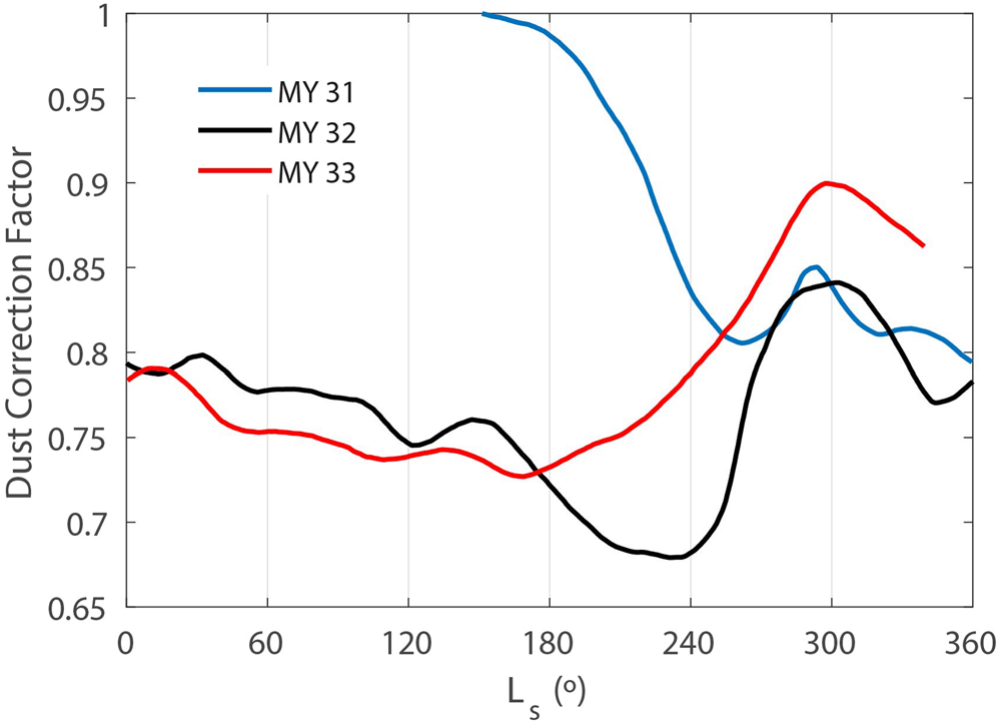
Temporal evolution of the Dust Correction Factor obtained for the UVE channel of the MSL REMS UV sensor during Martian Year 31 (blue), 32 (black) and 33 (red). Uncertainties (for clarity not included here; see methods) are shown in Supplementary Fig. S2. The mean value of the uncertainties is ± 0.025, with a maximum value of 0.052.

We have quantified dust accumulation on the REMS UV sensor by calculating a dust correction factor (DCF), defined as the fraction of the incoming UV radiation at the surface that reaches the photodiode through dust accumulated on the sensor, with respect to the fraction at the beginning of the mission (see methods). A DCF value equal to 1 indicates no additional attenuation of the radiation measured by the sensor while a value of 0.7 indicates that only 70% of the incoming radiation reaches the photodiode. Figure 2 shows the temporal evolution of the DCF obtained for the UVE (300–350 nm) channel during MY 31, 32 and 33. The UVE channel was selected because it has the best overall correlation with the other five channels (the sum of the five correlation coefficients is the highest, as shown in Supplementary Fig. S1) and because this channel matches the 320 nm channel of the Mars Color Imager of the Mars Reconnaissance Orbiter, from which the dust refractive indices used in our radiative transfer model were obtained^22^ (therefore, the accuracy of the DCF for this particular channel is expected to be higher than for the mean of the six channels). Nonetheless, as shown in Supplementary Fig. S1, the six channels show high values for the sum of their correlation coefficients, indicating the robustness of the results.

The DCF values indicate that the amount of dust accumulated on the sensor does not increase continuously throughout the mission, and that dust cleaning events prevent DCF values to fall well below ~ 0.7 while also producing significant temporal variability. At the beginning of the mission in MY 31, an abrupt decrease in the DCF (caused by dust accumulation) was observed until L_s_ ~ 260° (first ~ 180 sols). Then, the DCF increased (caused by dust removal) until L_s_ ~ 290°, followed by a decrease that persisted throughout the remainder of the perihelion season (until L_s_ = 360°). In MY 32 and 33, seasonal trends of DCF are qualitatively similar, but with some interannual variability in the duration and intensity of the cleaning events. In particular, DCF increased in MY 32 between L_s_ ~ 230°–300°, while in MY 33 DCF increased between L_s_ ~ 170°–300°. Values of the DCF were higher (indicating a cleaner sensor) at the end of the MY 33 cleaning season than at a similar time on MY 32. Then, the DCF decreased until the end of the aphelion season (L_s_ ~ 300°–180°).

The REMS UV sensor contains seven samarium-cobalt magnetic rings, six of them located around each photodiode and an additional ring located at the center of the six photodiodes^16^. These magnets are designed to deflect magnetic dust similarly to the sweep magnet of the Magnetic Properties Experiment on the MER^23^; this dust deflection effect is shown in Fig. 1.

The magnets can affect the results in two ways. First, analyses of images of the MER sweep magnet show that the circular area inside the magnetic ring is particularly clean^14,24,25^, indicating that the fraction of dust on Mars that is nonmagnetic or weakly magnetic is small. Therefore, the amount of dust deposited on the REMS photodiodes is expected to be smaller than if magnets were not present, with the DCF representing an upper bound for values on other parts of the rover deck. Second, the dust on the MER magnets can be classified into a dark and a bright fraction, with the grains of the bright fraction being smaller and less magnetic than those of the dark fraction^14,26,27^. Since dust particle effective radii at Gale changes with season^28,29^, the DCF could also be affected by seasonal variations in dust magnetic properties caused by changes in particle size.

We also note that the annual amplitude of the daily maximum values of REMS UV measurements has not decreased during the analyzed period (it has even increased in MY 33 with respect to MY 32 (not shown)), indicating that possible changes in sensitivity are insignificant for our conclusions on the seasonal and interannual variations of the DCF.

Our results support previous estimations of dust accumulation and removal from *in situ* measurements at other locations^13,14,24,30–34^. Analyses of data from the MER solar panels indicated that dust was removed from the solar panel of the Spirit rover between L_s_ = 170°–300°, with some interannual variability in duration, occurrence and intensity^13,14^. Also, analyses of the MER Pancam calibration targets indicated that there are periods of dust deposition and removal at both the Opportunity and Spirit locations, but that in general there are two peaks in dust deposition on Opportunity, one at L_s_ = 45° and a more irregular peak falling in the Spirit dust removal season^34^. MER results are in excellent agreement with our findings at the MSL location, with the seasonal pattern being more similar to the one at the Spirit location. In addition, our results are consistent with dust deposition and lifting patterns predicted by numerical models^9–12,35^.

### Dust Lifting and Deposition Mechanisms

**Figure 3:**
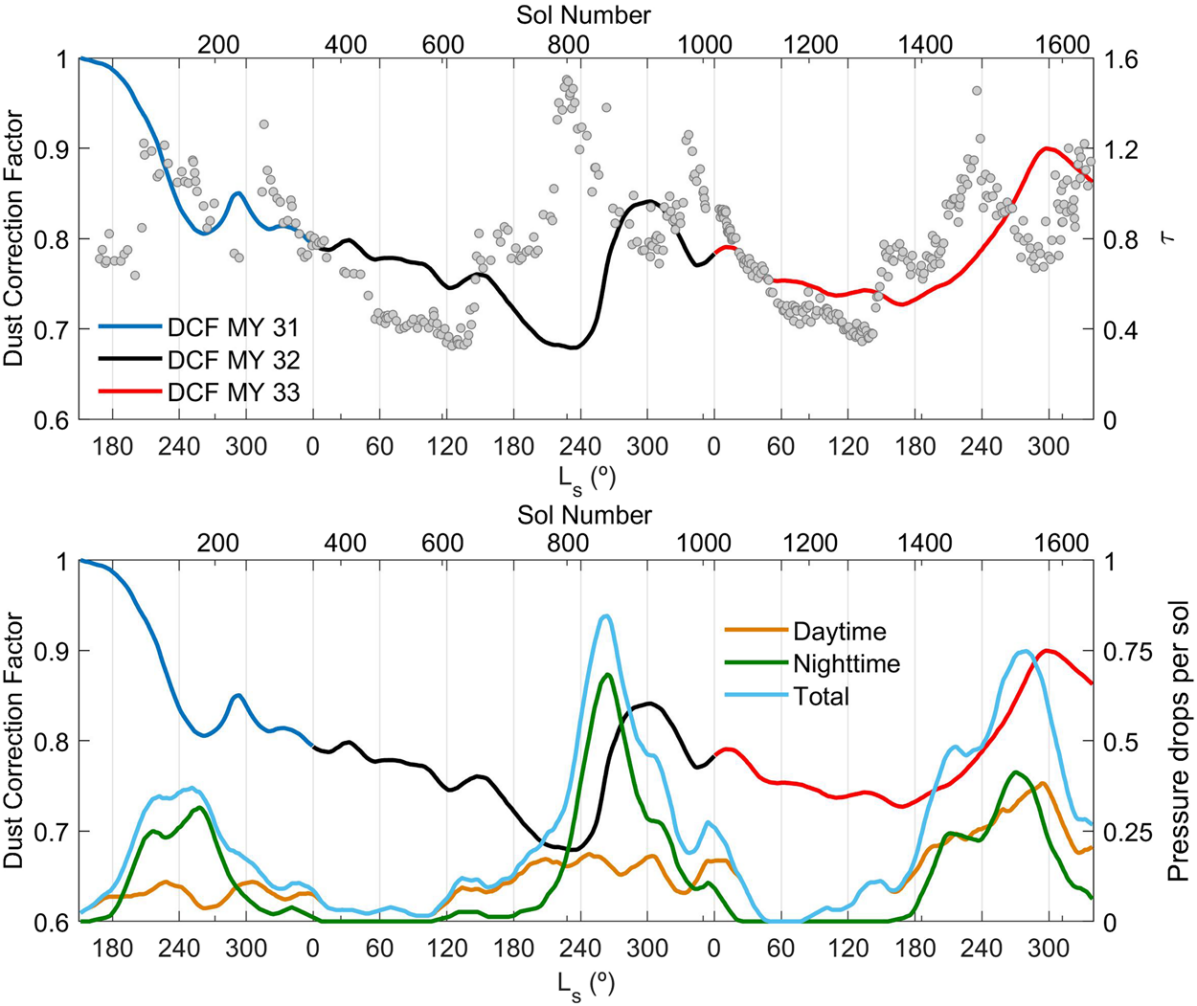
(Top) Temporal evolution of the DCF (with colors for each MY as in Fig. 2) and aerosol opacity (gray) during the first 1648 sols of the MSL mission. (Bottom) Temporal evolution of the DCF and daytime (dark orange), nighttime (dark green) and total (dark cyan) number of detected pressure drops above 0.5 Pa per sol (see text for details on pressure drop selection).

We have used atmospheric opacities retrieved from Mastcam images and REMS pressure measurements to shed light on the mechanisms driving the variability of the DCF shown in Fig. 2. The top panel of Fig. 3 compares the temporal evolution of DCF to that of atmospheric aerosol opacity. During the low opacity season (sols 400–600 and 1100–1300), decreases in the amount of suspended dust are correlated with decreases in DCF (periods in which the amount of dust accumulated on the sensor increases). This suggests that during the aphelion season, gravitational settling of atmospheric dust is responsible for both the accumulation of dust on the surface and the cleaning of the atmosphere. In contrast, these two quantities are not correlated during the high opacity season, when large quantities of dust are ejected into the atmosphere. Each year, dust is removed from the sensor when the atmospheric aerosol opacity is relatively high (sols 800–900 and 1400–1550), indicating that at this time of the year dust lifting exceeds gravitational settling. The absence of a time lag between the increase in the DCF and the annual maximum opacity value, especially in MY 32, suggests that dust transported from outside the crater plays an important role in the increase in opacity at the MSL location. This is consistent with the strong and persistent winds at Gale during the high opacity season^36^.

The top panel of Fig. 3 indicates that estimation of atmospheric opacity alone is not enough for assessing or predicting the energy generated by solar-powered spacecraft, because the net dust accumulation also needs to be estimated. For example, the attenuation of solar radiation in MY 32 caused by dust accumulation is up to 2.5 times greater than that caused by airborne dust throughout most of the year, except between L_s_ ~ 240–360°, when both contributions are similar (Supplementary Fig. S3). Since the REMS UV photodiodes are surrounded by magnetic rings, dust accumulation is expected to have an even larger impact on solar panels, which lack this partial protection.

The bottom panel of Fig. 3 compares the temporal evolution of the DCF to that of the number of pressure drops per sol larger than 0.5 Pa. These pressure drops are indicative of the passage of convective vortices^37,38^ and of turbulent eddies induced by wind shear^38^. The nighttime pressure drops appear to be caused by the interactions between the global atmospheric circulation and the local circulation at Gale, which results in strong, flushing northerly winds, and in an enhancement of mountain wave activity; the accelerating winds increase the vertical wind shear, which leads to enhanced turbulence^36,39^. Since the duration of the MSL pressure measurements can vary from sol to sol^40^, we have only considered the pressure drops detected during the first six minutes of each hour, which are available throughout the mission. Values of the number of pressure drops per sol were averaged in the same way as the DCF values (see methods). The temporal evolutions of the number of daytime, nighttime and total pressure drops are shown in dark orange, dark green and dark cyan, respectively. The frequency of pressure drops is low during the aphelion season when the DCF decreases (dust deposition), indicating that gravitational settling is the dominant process at this time of the year. In contrast, the frequency of pressure drops is maximum during the dust removal season, suggesting that the lifting and removal of dust from the sensor is caused by surface winds and convective vortices.

We have analyzed the frequency of these pressure drops as a function of local time to investigate the relative contribution of convective vortices and surface winds to the cleaning of the UV sensor, indicated by increases in the DCF. We observe that the daily frequency of such pressure drops has a bimodal distribution, with one peak around local noon, consistent with previous meteorological observations and simulations^12,37,41^ and with the distribution of detected dust lifting in MSL Navcam and Mastcam images^42,43^ and a second peak around midnight (Supplementary Fig. S4). Following this bimodal distribution, we have used the MarsWRF nested mesoscale model to simulate the temporal evolution of Dust Devil Activity (DDA) at noon and surface wind stress at midnight. Analyses of simulations of the wind stress as a function of local time for both the aphelion and perihelion season (not shown) indicate that the overall results and conclusions do not change if the wind stress at the nighttime peak in pressure drops is used in the analysis. The MarsWRF simulations are as described in Newman *et al*.^21^ using vertical grid B, with the atmospheric dust distribution prescribed using the MGS dust scenario of the Mars Climate Database^44^. The results shown in this section are from the innermost nest of the model, which has a spatial resolution of ~490 m, and are interpolated to the location of the rover in the relevant sol. The calculation of these quantities in the MarsWRF model is explained in the methods, and further details are provided by Newman and Richardson^20^.

**Figure 4:**
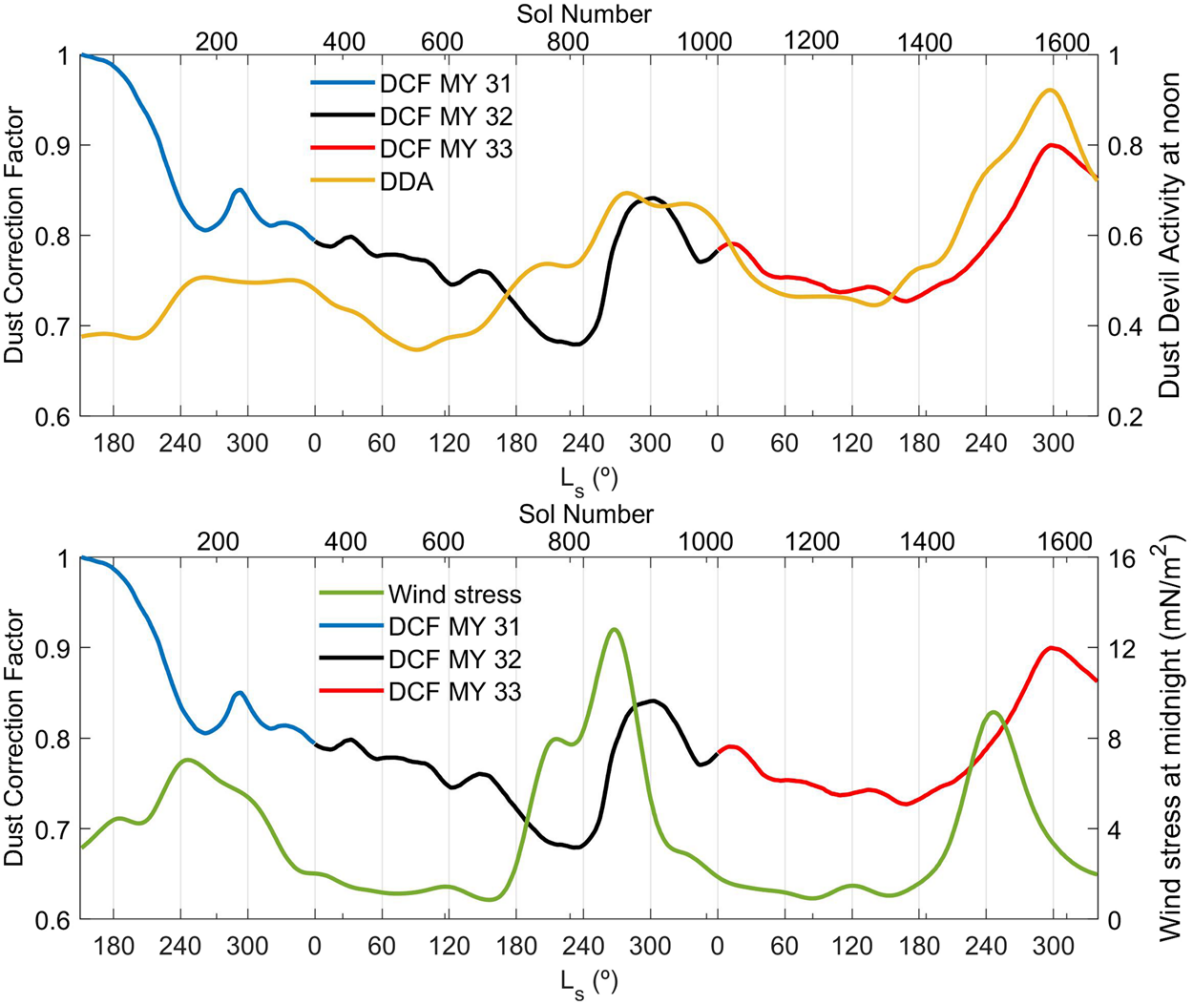
(Top) Temporal evolution of the DCF (blue, black and red) and DDA (in arbitrary units) at noon (light orange) as simulated by MarsWRF during the first 1648 sols of the MSL mission. (Bottom) Temporal evolution of the DCF and wind stress at midnight (light green) as simulated by MarsWRF during the first 1648 sols of the MSL mission.

Figure 4 compares the temporal evolution of the DCF to that of the simulated DDA at noon in the top panel, and to that of the simulated wind stress at midnight in the bottom panel. DDA appears to correlate with DCF, with high DDA values during the dust removal season, while nighttime wind stress peaks during the main dust lifting events. These peaks in wind stress are associated with strong regional winds (due to the global circulation reinforced by dichotomy boundary flows creating a ‘downslope wind storm’) entering the crater over the N and NW rims. The increase in DCF during MY 33 follows a similar trend to that of DDA at noon, whereas the dust removal season in MY 32 is shorter and the increase in DCF is more abrupt than in MY 33, with a higher resemblance to the peak in nocturnal wind stress in MY 32. These findings suggest that nighttime winds (understood as their total component, as analyses of the results do not allow to address separately the roles of steady winds and turbulent eddies) have a more prominent role in removing dust from the UV sensor in MY 32 than in MY 33, and that daytime convective vortices play a larger role in dust removal in MY 33 than in MY 32. This suggestion is further supported by higher DDA values in MY 33 than in MY 32 (the frequency of daytime pressure drops above 0.5 Pa measured during the dust removal season of MY 33 is twice its value during that of MY 32), and by higher nighttime wind stress values in MY 32 than in MY 33. This behavior is consistent with stronger dust devil activity in MY 33 as Curiosity climbs the slope of Aeolis Mons, a region where the differences between surface and air temperatures are larger due to a lower ground thermal inertia^45,46^ and where drag velocities are stronger due to daytime upslope winds on some portions of the slopes (simulated wind stresses at noon are not well correlated with the DCF (not shown), suggesting that convective vortices play a more important role than winds at those times). It is also consistent with weaker nighttime wind stresses in MY 33 as the rover moves towards the slopes of Aeolis Mons which are further from the northern rim, from which strong winds flow into the crater at night during the time of the year centered around L_s_ = 270° ^36^, the season of the strongest cleaning events.

## Conclusions

We have quantified the seasonal and interannual variability of dust accumulation and removal on a Mars surface asset using measurements of solar UV radiation by the Curiosity rover. We have shown that dust accumulation has a repeatable seasonal cycle with some interannual variability. Dust is removed during the perihelion season until L_s_ ~ 300° and is deposited from the end of the perihelion season throughout the full aphelion season (L_s_ ~ 300°–180°).

Analyses of MER solar panel data^13,14^, particularly of Spirit, are in excellent agreement with our results from MSL UVS data, suggesting that cleaning mechanisms appear to be ubiquitous on Mars, at least at these areas in which data is currently available, although there are some differences in intensity, occurrence and duration.

We have used independent *in situ* measurements of aerosol opacities and pressure measurements, in combination with simulations by the MarsWRF mesoscale model, to investigate the mechanisms responsible for the variability of dust accumulation and removal. Gravitational settling of atmospheric dust is responsible for the accumulation of dust on the UV sensor during the aphelion season, whereas daytime convective vortices and nighttime winds are likely responsible for the seasonal cleaning during the perihelion season, with nighttime winds playing a more prominent role in MY 32 than in MY 33, and with daytime convective vortices having a larger role in MY 33 than in MY 32. This is consistent with the rover moving from a location with a local topography that favors wind-induced turbulent eddies in MY 32 to higher altitude locations with lower ground thermal inertia and stronger daytime upslope winds that favor dust devil activity in MY 33.

Our results allow a better tuning of the estimates of dust lifting and gravitational settling in Martian models. Moreover, they provide important information for the operations of future solar powered missions, such as the InSight lander, en route to Mars. First, if high frequency pressure measurements are available but solar radiation measurements are not, pressure perturbations can be used as a proxy to identify dust removal events. Second, dust accumulation does not increase continuously after a few months, indicating that solar energy can be used to power multiannual Mars missions. Third, examination of dust lifting mechanisms from MarsWRF suggest that the location of an asset on the surface of Mars is an important factor controlling dust cleaning and should be incorporated in analyses of future solar-powered mission scenarios. Finally, dust accumulation in general has a greater impact on incoming solar fluxes than suspended dust throughout the year. Therefore, variations in the amount of dust accumulated on solar panels should be considered in spacecraft design and operations.

## Methods

### Calculation of the Dust Correction Factor

We developed a technique to quantify the effect of dust accumulated on the REMS UV sensor based on the calculation of a dust correction factor (DCF) parameter that depends only on the amount of dust accumulated on the sensor. The DCF is defined as the fraction of the incoming UV radiation at the surface that reaches the photodiode (through dust accumulation on the sensor optics) with respect to that at the beginning of the mission, when the sensor was relatively clean.

To calculate DCF we selected photodiode output currents measurements of the six channels of the REMS UV sensor on sols on which the atmospheric opacity was retrieved from Mastcam images. To avoid measurements affected by shadows cast by the masthead and the mast of the rover, we discarded those measurements for which the masthead of the rover was not in its most typical position (azimuth ~−179° (looking forward) and elevation ~43° (1°/91° means pointing down/forward), allowing a margin of 0.01°), and those with a solar azimuth angle relative to the rover frame between −100° and 10° ^28^. We also discarded measurements performed between the initial and final times of each rover drive and those when the solar zenith angle was larger than 45° in order to minimize uncertainties in our retrievals. Then, for each selected sol, we calculated the average output current as a function of the position of the Sun and stored such averages in a grid with cells separated by 1° in solar zenith angle and 5° in solar azimuth angle. For each possible combination of two sols in chronological order, we selected the two cells that represent the same position of the Sun on both sols for which the sum of the output currents on both sols is the highest. This way, the signal-to-noise ratio is improved. For each of these two cells, we normalized its value by considering changes in the distance between the Sun and Mars and in the atmospheric opacity. This normalization was applied using the radiative transfer model COMIMART^47^. For the selected pair of sols, we calculated the ratio between the normalized value of the second sol and that of the first sol and stored such ratio in a table. This ratio represents the value of the DCF on the second sol assuming that on the first sol (considered as a reference) is equal to one.

For a given sol (hereinafter sol s), we searched all the sols used as a reference to obtain a relative DCF value for sol s (subset 1) and all the sols for which a relative DCF was calculated using sol s as a reference (subset 2). We scaled all the relative DCFs obtained using as a reference sols in subset 1 so that all the relative DCFs obtained for sol s are equal to 1. Then we scaled all the relative DCFs obtained using as a reference each sol in subset 2 by multiplying them by the relative DCF of the corresponding sol in subset 2. As an example, let us assume the following hypothetical scenario that considers 5 sols with relative DCFs. There are two relative DCFs for sol 3 obtained using sols 1 and 2 as references, and also a relative DCF for sol 4 and another relative DCF for sol 5 both obtained using sol 3 as a reference. If sol 3 is our sol s, then sols 1 and 2 belong to subset 1 and sols 4 and 5 belong to subset 2.

Among all the values of the relative DCFs normalized by the value on sol s, we searched for the one obtained with the smallest value of the solar zenith angle for each sol to minimize uncertainties. This way we obtain a temporal series of the DCF normalized to the value on sol s. We repeat this process for each sol with opacity retrievals. Then we normalized all the obtained temporal series and we obtained the temporal evolution of the DCF, normalized to the value on the first sol with Mastcam opacity retrievals. In order to improve the analysis of seasonal and interannual variations, results were smoothed by applying a weighting function with a triangular shape that reaches a value 0 at 50 sols from each sol and remains with that value beyond. We have observed that dust lifting and deposition on the sensor occur gradually and we have not detected sudden cleaning events as those observed at the Spirit site^34^ that could have been masked with the smoothing function. The mean and the standard deviations of the differences between the values of the DCF obtained before and after smoothing were calculated for each sol considering the values within 50 sols of it. The upper (lower) boundaries of the uncertainties were calculated from the standard deviations of the differences and, following a conservative approach, adding (subtracting) the mean values of the differences when these were positive (negative).

### Calculation of the Dust Devil Activity and wind stress

Dust devils are treated in the MarsWRF model used in this study as convective heat engines, since this approach leads to the best match to the seasonal evolution of the background amount of dust^20^. In this scheme, the Dust Devil Activity is defined as the product of the surface sensible heat flux times the thermodynamic efficiency of the dust devil convective engine; the first is proportional to the difference between ground temperature and near surface air temperature, and the second depends on the thickness of the boundary layer^48^. This scheme allows us to predict vortex activity based on the large-scale meteorological variables, but it is not based on any sub-grid scale parametrization of turbulent eddies, nor on explicit Large Eddy Simulations of them. Wind stress is calculated from the total (including all the components) winds predicted by the ~490 m resolution mesoscale model using a surface layer parametrization that follows the Monin - Obukhov scheme^49^. Additional details were provided by Newman and Richardson^20^.

### Calculation of the attenuation caused by suspended dust

This attenuation, shown in Supplementary Fig. S3, represents the fraction of the incoming radiation at the top of the Martian atmosphere that is absorbed or scattered back to space by suspended dust particles, and therefore does not reach the surface. It depends on the amount of suspended dust, on the time of the day and on the radiative properties of the particles. We calculated it using our radiative transfer model COMIMART^47^. The sources of uncertainties are potential inaccuracies in the estimations of aerosol abundance and properties, and biases in the calculation of solar fluxes using the delta-Eddington approximation^50^. Uncertainties in dust opacity have been computed as explained for the Dust Correction Factor, and adding to them 0.03, which is the typical uncertainty in Mastcam opacities^5,17^. Then, uncertainties in the attenuation by suspended dust have been computed by simulating the attenuations with COMIMART using the upper and lower boundaries of the opacities. Finally, inaccuracies in aerosol properties (mainly attributed to uncertainties in the particle size distribution, dust refractive indices and particle shape) can also affect the values of the attenuation. For a fixed opacity, an overestimation of particle size would lead to an underestimation of the attenuation by suspended dust^28^. Therefore, values of the attenuation due to suspended dust are expected to be slightly overestimated during the low opacity season (when particles are smaller) and to be slightly underestimated around the enhanced opacity events (when particles are larger)^28,29^. In order to quantify this effect, we performed simulations with COMIMART for two extreme scenarios, the first one with a high opacity value of 1.2 and the upper bound of particle size (effective radius of 2 μm), and the second one with a low opacity value of 0.4 and the lower bound of particle size (effective radius of 0.5 μm). Assuming a solar zenith angle of 45°, suspended dust attenuation would be underestimated by ~5% for the first scenario and would be overestimated by ~25%. Therefore, the main conclusions regarding the importance of dust accumulation would remain unaltered, since suspended dust attenuation is similar to deposited dust attenuation during the high opacity season, and deposited dust attenuation is larger than suspended dust deposition during the rest of the year. Finally, biases in solar fluxes at the surface are generally around 1% or less^47^.

## Electronic supplementary material

Supplementary Information

## References

[1] Smith MD (2004). Interannual variability in TES atmospheric observations of Mars during 1999–2003. Icarus.

[2] Smith MD (2009). THEMIS observations of Mars aerosol optical depth from 2002–2008. Icarus.

[3] Montabone L (2015). Eight-year climatology of dust optical depth on Mars. Icarus.

[4] Colburn DS, Pollack JB, Haberle RM (1989). Diurnal variations in optical depth at Mars. Icarus.

[5] Lemmon MT (2015). Dust aerosol, clouds, and the atmospheric optical depth record over 5 Mars years of the Mars Exploration Rover mission. Icarus.

[6] Martínez G. M., Newman C. N., De Vicente-Retortillo A., Fischer E., Renno N. O., Richardson M. I., Fairén A. G., Genzer M., Guzewich S. D., Haberle R. M., Harri A.-M., Kemppinen O., Lemmon M. T., Smith M. D., de la Torre-Juárez M., Vasavada A. R. (2017). The Modern Near-Surface Martian Climate: A Review of In-situ Meteorological Data from Viking to Curiosity. Space Science Reviews.

[7] Haberle RM, Conway BL, Pollack JB (1982). Some effects of global dust storms on the atmospheric circulation of Mars. Icarus.

[8] Read, P. L. & Lewis, S. R. The Martian Climate Revisited: Atmosphere and Environment of a Desert Planet (Springer-Verlag, Berlin, 2004).

[9] Newman Claire E., Lewis Stephen R., Read Peter L., Forget François (2002). Modeling the Martian dust cycle 2. Multiannual radiatively active dust transport simulations. Journal of Geophysical Research: Planets.

[10] Newman Claire E., Lewis Stephen R., Read Peter L., Forget François (2002). Modeling the Martian dust cycle 2. Multiannual radiatively active dust transport simulations. Journal of Geophysical Research: Planets.

[11] Basu, S., Richardson, M. I. & Wilson, R. J. Simulation of the Martian dust cycle with the GFDL Mars GCM. *Journal of Geophysical Research: Planets*, **109**(E11) (2004).

[12] Kahre, M. A., Murphy, J. R. & Haberle, R. M. Modeling the Martian dust cycle and surface dust reservoirs with the NASA Ames general circulation model. *J*. *Geophys*. *Res*. *Planets*, **111**(E6) (2006).

[13] Stella, P. M. & Herman, J. A. The Mars surface environment and solar array performance. In *Photovoltaic Specialists Conference (PVSC)*, *2010 35th IEEE* (pp. 002631–002635) (2010).

[14] Vaughan AF (2010). Pancam and Microscopic Imager observations of dust on the Spirit Rover: Cleaning events, spectral properties, and aggregates. Mars.

[15] Grotzinger JP (2012). Mars Science Laboratory Mission and Science Investigation. Space Sci. Rev..

[16] Gómez-Elvira J (2012). REMS: The Environmental Sensor Suite for the Mars Science Laboratory Rover. Space Sci. Rev..

[17] Smith MD, Zorzano M-P, Lemmon M, Martín-Torres J, Mendaza de Cal T (2016). Aerosol optical depth as observed by the Mars Science Laboratory REMS UV photodiodes. Icarus.

[18] Edgett KS (2012). Curiosity’s Mars hand lens imager (MAHLI) investigation. Space science reviews.

[19] Richardson, M. I., Toigo, A. D. & Newman, C. E. PlanetWRF: A general purpose, local to global numerical model for planetary atmospheric and climate dynamics. *Journal of Geophysical Research: Planets*, **112** (E9) (2007).

[20] Newman CE, Richardson MI (2015). The impact of surface dust source exhaustion on the martian dust cycle, dust storms and interannual variability, as simulated by the MarsWRF General Circulation Model. Icarus.

[21] Newman CE (2017). Winds measured by the Rover Environmental Monitoring Station (REMS) during the Mars Science Laboratory (MSL) rover’s Bagnold Dunes Campaign and comparison with numerical modeling using MarsWRF. Icarus.

[22] Wolff MJ, Clancy RT, Goguen JD, Malin MC, Cantor BA (2010). Ultraviolet dust aerosol properties as observed by MARCI. Icarus.

[23] Madsen, M. B. *et al*. Magnetic properties experiments on the Mars Exploration Rover mission. *Journal of Geophysical Research: Planets*, **108**(E12) (2003).

[24] Kinch, K. M. *et al*. Dust deposition on the Mars Exploration Rover Panoramic Camera (Pancam) calibration targets. *Journal of Geophysical Research: Planets*, **112**(E6) (2007).

[25] Bertelsen P (2004). Magnetic properties experiments on the Mars Exploration Rover Spirit at Gusev crater. Science.

[26] Kinch KM (2006). Preliminary analysis of the MER magnetic properties experiment using a computational fluid dynamics model. Planetary and Space Science.

[27] Madsen, M. B. *et al*. Overview of the magnetic properties experiments on the Mars Exploration Rovers. *Journal of Geophysical Research: Planets*, **114**(E6) (2009).

[28] Vicente-Retortillo A, Martínez GM, Renno NO, Lemmon MT, de la Torre-Juárez M (2017). Determination of dust aerosol particle size at Gale Crater using REMS/UVS and Mastcam measurements. Geophys. Res. Lett..

[29] McConnochie TH (2018). Retrieval of water vapor column abundance and aerosol properties from ChemCam passive sky spectroscopy. Icarus.

[30] Guinness EA, Leff CE, Arvidson RE (1982). Two Mars years of surface changes seen at the Viking landing sites. Journal of Geophysical Research: Solid Earth.

[31] Johnson JR, Grundy WM, Lemmon MT (2003). Dust deposition at the Mars Pathfinder landing site: Observations and modeling of visible/near-infrared spectra. Icarus.

[32] Arvidson RE (2004). Localization and physical property experiments conducted by Opportunity at Meridiani Planum. Science.

[33] Drube, L. *et al*. Magnetic and optical properties of airborne dust and settling rates of dust at the Phoenix landing site. *Journal of Geophysical Research: Planets*, **115** (E5) (2010).

[34] Kinch KM (2015). Dust deposition on the decks of the Mars Exploration Rovers: 10 years of dust dynamics on the Panoramic Camera calibration targets. Earth and Space Science.

[35] Kahre, M. A. *et al*. The Mars Dust Cycle. In: Haberle, R. M., Clancy, R.T., Forget, F., Smith, M. D. and Zurek, R. W. (Editors). *The Atmosphere and Climate of Mars*, 295-337 (Cambridge University Press, 2017).

[36] Rafkin SC (2016). The meteorology of Gale Crater as determined from Rover Environmental Monitoring Station observations and numerical modeling. Part II: Interpretation. Icarus.

[37] Kahanpää H (2016). Convective vortices and dust devils at the MSL landing site: Annual variability. Journal of Geophysical Research: Planets.

[38] Ordonez-Etxeberria I, Hueso R, Sánchez-Lavega A (2018). A systematic search of sudden pressure drops on Gale crater during two Martian years derived from MSL/REMS data. Icarus.

[39] Pla-Garcia J (2016). The meteorology of Gale crater as determined from rover environmental monitoring station observations and numerical modeling. Part I: Comparison of model simulations with observations. Icarus.

[40] Gómez-Elvira J (2014). Curiosity’s rover environmental monitoring station: Overview of the first 100 sols. J. Geophys. Res. Planets.

[41] Murphy James R., Nelli Steven (2002). Mars Pathfinder convective vortices: Frequency of occurrence. Geophysical Research Letters.

[42] Lemmon, M. T. *et al*. Dust devil activity at the Curiosity Mars rover field site. In Lunar and Planetary Science Conference (Vol. 48) (2017).

[43] Kahanpää, H. *et al*. Martian Dust Devils Observed Simultaneously by Imaging and by Meteorological Measurements. In Lunar and Planetary Science Conference (Vol. 49) (2018).

[44] Montmessin, F., Forget, F., Rannou, P., Cabane, M. & Haberle, R. M. Origin and role of water ice clouds in the Martian water cycle as inferred from a general circulation model. *Journal of Geophysical Research: Planets*, **109**(E10) (2004).

[45] Thomson BJ (2011). Constraints on the origin and evolution of the layered mound in Gale Crater, Mars using Mars Reconnaissance Orbiter data. Icarus.

[46] Toigo AD, Lee C, Newman CE, Richardson MI (2012). The impact of resolution on the dynamics of the martian global atmosphere: Varying resolution studies with the MarsWRF GCM. Icarus.

[47] Vicente-Retortillo Á, Valero F, Vázquez L, Martínez GM (2015). A model to calculate solar radiation fluxes on the Martian surface. Journal of Space Weather and Space Climate.

[48] Rennó NO, Burkett ML, Larkin MP (1998). A simple thermodynamical theory for dust devils. Journal of the Atmospheric Sciences.

[49] Jiménez PA, Dudhia J (2012). Improving the representation of resolved and unresolved topographic effects on surface wind in the WRF model. Journal of Applied Meteorology and Climatology.

[50] Joseph JH, Wiscombe WJ, Weinman JA (1976). The delta-Eddington approximation for radiative flux transfer. Journal of the Atmospheric Sciences.

