# Supplementary material for: Seasonal Deposition and Lifting of Dust on Mars as Observed by the Curiosity Rover
Source: Sci Rep. 2018 Dec 4;8:17576. doi: 10.1038/s41598-018-35946-8 (PMC6279765; doi:10.1038/s41598-018-35946-8)
Supplement: Supplementary file 1 — Supplementary Information [file 41598_2018_35946_MOESM1_ESM.pdf]

Seasonal Deposition and Lifting of Dust on Mars as Observed by the Curiosity Rover

Á. Vicente-Retortillo[1*], G. M. Martínez[1], N. Renno[1], C. E. Newman[2], I. Ordonez-Etxeberria[3], M. T. Lemmon[4], M. I. Richardson[2], R. Hueso[3] and A. Sánchez-Lavega[3]

[1]Department of Climate and Space Sciences and Engineering, University of Michigan, Ann Arbor, MI, USA.

[2]Aeolis Research, Pasadena, CA, USA

[3]Departamento de Física Aplicada I, Universidad del País Vasco, Bilbao, Spain.

[4]Department of Atmospheric Sciences, Texas A&M University, College Station, TX, USA.

[*]Corresponding author: alvarode@umich.edu

Supplementary Information

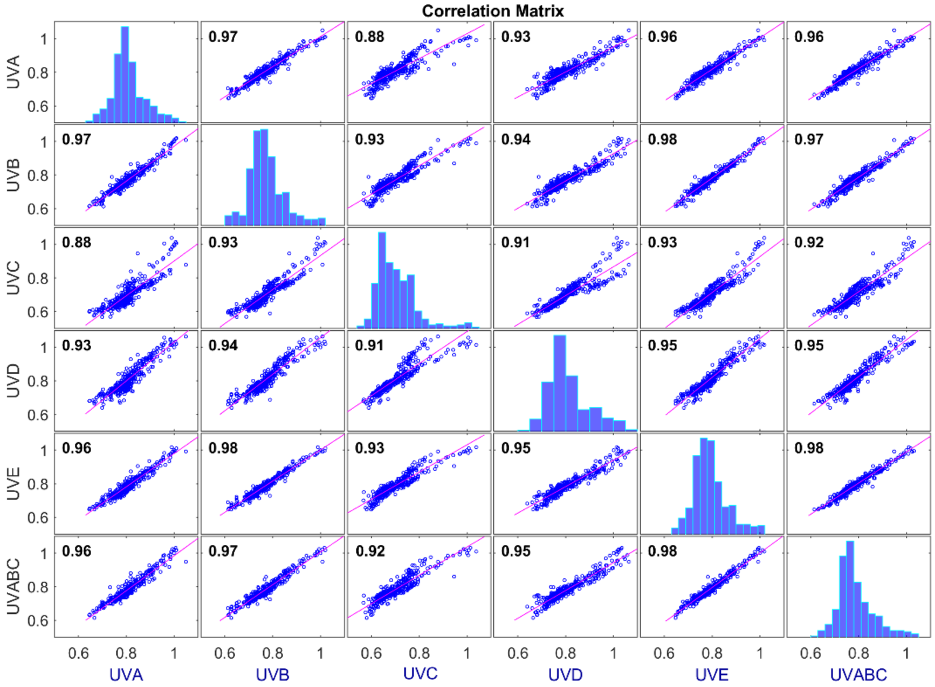

Fig. S1. Correlations among Dust Correction Factors for each pair of channels. Histograms for each channel are shown in the diagonal of the matrix of plots. The value indicated in each correlation plot is the correlation coefficient. There is an excellent correlation between the values of the different channels, indicating the robustness of the results.

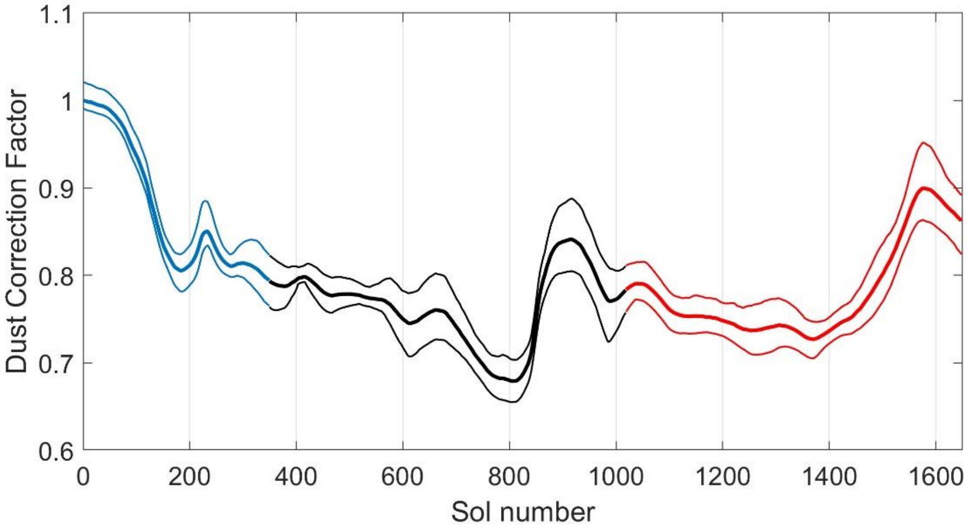

Fig. S2. Temporal evolution of the DCF (thick line with colors as in Fig. 1) and its uncertainties (thin lines), calculated as explained in the methods.

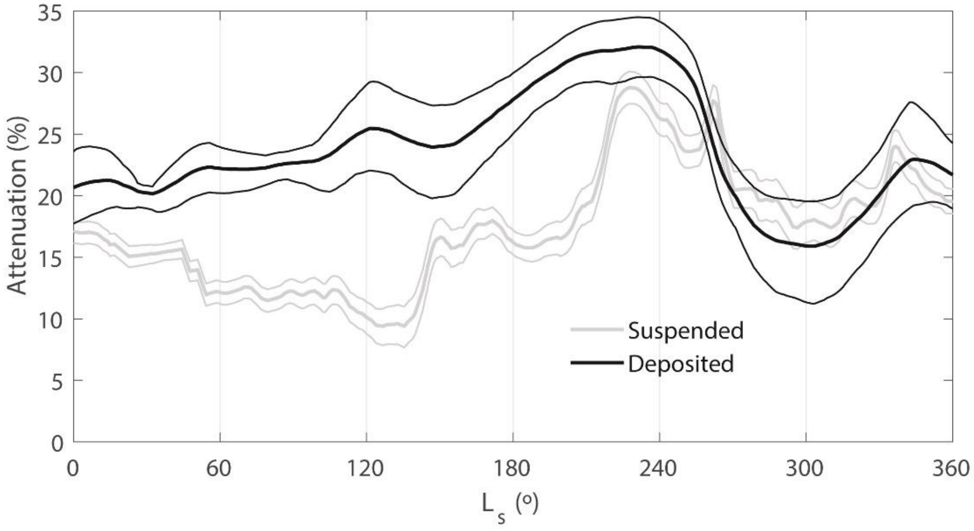

Fig. S3. Attenuation of incoming radiation in MY 32 caused by accumulation (black) and suspended (gray) dust. Thin lines represent uncertainties (see methods).

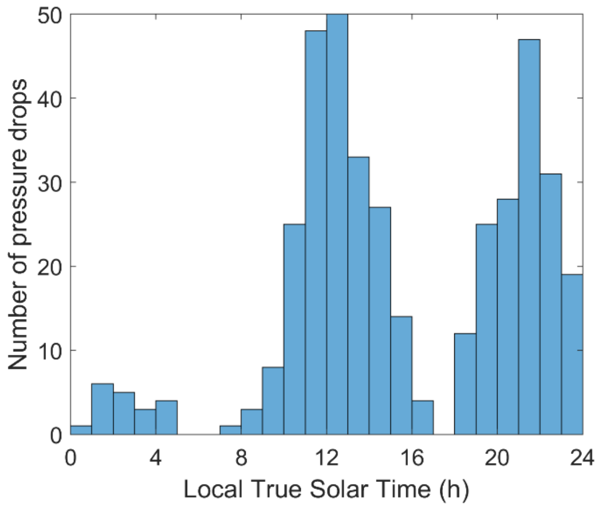

Fig. S4. Number of pressure drops above 0.5 Pa measured by REMS during the first six minutes of each hour as a function of the time of the day.
